# Novel Short-Chain Quinones to Treat Vision Loss in a Rat Model of Diabetic Retinopathy

**DOI:** 10.3390/ijms22031016

**Published:** 2021-01-20

**Authors:** Abraham Daniel, Dino Premilovac, Lisa Foa, Zikai Feng, Krupali Shah, Qianyi Zhang, Krystel L. Woolley, Nicole Bye, Jason A. Smith, Nuri Gueven

**Affiliations:** 1 School of Pharmacy and Pharmacology, University of Tasmania, Hobart, TAS 7005, Australia; Abraham.Daniel@utas.edu.au (A.D.); zikai.feng@utas.edu.au (Z.F.); krupali.shah@utas.edu.au (K.S.); qzhang17@utas.edu.au (Q.Z.); Nicole.Bye@utas.edu.au (N.B.); 2 Tasmanian School of Medicine, University of Tasmania, Hobart, TAS 7005, Australia; Dino.Premilovac@utas.edu.au (D.P.); Lisa.Foa@utas.edu.au (L.F.); 3 School of Psychological Science, University of Tasmania, Hobart, TAS 7005, Australia; 4 School of Natural Sciences-Chemistry, University of Tasmania, Hobart, TAS 7005, Australia; krystel.woolley@utas.edu.au (K.L.W.); jason.smith@utas.edu.au (J.A.S.)

**Keywords:** diabetic-retinopathy, mitochondria, idebenone, short-chain quinones, elamipretide

## Abstract

Diabetic retinopathy (DR), one of the leading causes of blindness, is mainly diagnosed based on the vascular pathology of the disease. Current treatment options largely focus on this aspect with mostly insufficient therapeutic long-term efficacy. Mounting evidence implicates mitochondrial dysfunction and oxidative stress in the central etiology of DR. Consequently, drug candidates that aim at normalizing mitochondrial function could be an attractive therapeutic approach. This study compared the mitoprotective compounds, idebenone and elamipretide, side-by-side against two novel short-chain quinones (SCQs) in a rat model of DR. The model effectively mimicked type 2 diabetes over 21 weeks. During this period, visual acuity was monitored by measuring optokinetic response (OKR). Vision loss occurred 5–8 weeks after the onset of hyperglycemia. After 10 weeks of hyperglycemia, visual function was reduced by 65%. From this point, the right eyes of the animals were topically treated once daily with the test compounds. The left, untreated eye served as an internal control. Only three weeks of topical treatment significantly restored vision from 35% to 58–80%, while visual acuity of the non-treated eyes continued to deteriorate. Interestingly, the two novel SCQs restored visual acuity better than idebenone or elamipretide. This was also reflected by protection of retinal pathology against oxidative damage, retinal ganglion cell loss, reactive gliosis, vascular leakage, and retinal thinning. Overall, mitoprotective and, in particular, SCQ-based compounds have the potential to be developed into effective and fast-acting drug candidates against DR.

## 1. Introduction

Diabetic retinopathy (DR) is a microvascular, neurodegenerative disease, as a complication of chronic hyperglycemia, and clinically manifests as progressive vision loss [1]. In the early stages of DR, most patients remain asymptomatic, but as the disease progresses, visual symptoms such as eye floaters, blurred vision, and visual distortion manifest [2].

Neuronal degeneration and microvascular damage are widely reported in DR [3], with neuronal degeneration possibly preceding vascular changes [4]. Despite this, the main therapeutic focus aims at counteracting the vascular changes of DR such as excessive angiogenesis, increased permeability, retinal inflammation, as well as increased intraocular pressure (IOP) [1]. At present, intravitreal administration of corticosteroids and antivascular endothelial growth factor (anti-VEGF) therapy, laser photocoagulation, and vitrectomy are used to treat DR, depending on the pathology and stage of the disease [5]. While treatments such as intravitreal steroids and anti-VEGF agents represent successful first-line treatments against inflammation and angiogenesis associated with DR [6,7], their invasive mode of administration remains unattractive [5,8]. In addition, most therapies are only beneficial in the progressive stage of DR [5,9], with fewer treatment options for nonproliferative DR [5]. Similarly, therapies that focus on the neuronal pathology of DR need to be explored further [8]. Hence, there is an urgent need for new approaches to counteract the vision loss associated with DR.

Mounting evidence implicates mitochondrial dysfunction as central to the etiology of DR. Recent studies have suggested that mitochondrial dysfunction and retinal ganglion cell (RGC) damage occur before vascular changes can be detected [8,10]. It has been proposed that mitochondria-derived reactive oxygen species (ROS) initiate additional DR pathologies such as mitochondrial DNA (mtDNA) damage, increased caspase activation, and release of inflammatory mediators [11,12], which further accelerate mitochondrial ROS production; while increased caspase activity and proinflammatory mediators directly lead to RGC loss [11,12]. Current therapies largely ignore mitochondrial dysfunction as central to the initiation and progression of DR. 

Currently, the only marketed mitoprotective therapy is idebenone, which is commercially available as Raxone^®^ for the treatment of Leber’s hereditary optic neuropathy (LHON), in Europe. LHON is the most common inherited mitochondrial disease caused by mutations in complex I of the mitochondrial respiratory chain [13]. According to the reversible redox chemistry of the quinone head, idebenone is associated with antioxidant activity and donates electrons to complex III of the mitochondrial respiratory chain, which increases ATP levels in the presence of a dysfunctional mitochondrial complex I [13,14,15]. Both activities are thought to be responsible for the reported rescue of visual acuity and normalized retinal pathology in LHON patients [13]. 

Several other mitoprotective drug candidates are currently in clinical development for a number of ophthalmological and neurodegenerative disorders [13]. However, with the exception of elamipretide (MTP-131) [8], none of these potential mitochondrial therapies have been trialed in preclinical models of DR. Elamipretide is a tetrapeptide that protects against cardiolipin peroxidation and has been reported to protect human retinal endothelial cells from high glucose-induced injury [8,16].

In the present study, we compared two novel mitoprotective short-chain quinones (SCQs) (#37 and #77) side-by-side against the mitoprotective drugs, idebenone and elamipretide, in a rat model of diabetes-induced vision loss [17]. The two cytoprotective SCQs had been previously identified in vitro by their significantly increased metabolic stability and cytoprotection against mitochondrial dysfunction as compared with the reference compound idebenone [18,19]. Elamipretide was included in the study as a reference compound that previously recovered visual acuity in a mouse model of diabetic retinopathy [8]. We hypothesize that mitoprotective compounds, in general, can counteract vision loss in DR and that the novel SCQs show increased therapeutic activity as compared with the reference compound idebenone.

## 2. Results

### 2.1. Effect of Test Compounds on Diabetes-Induced Vision Loss

**Figure 1:**
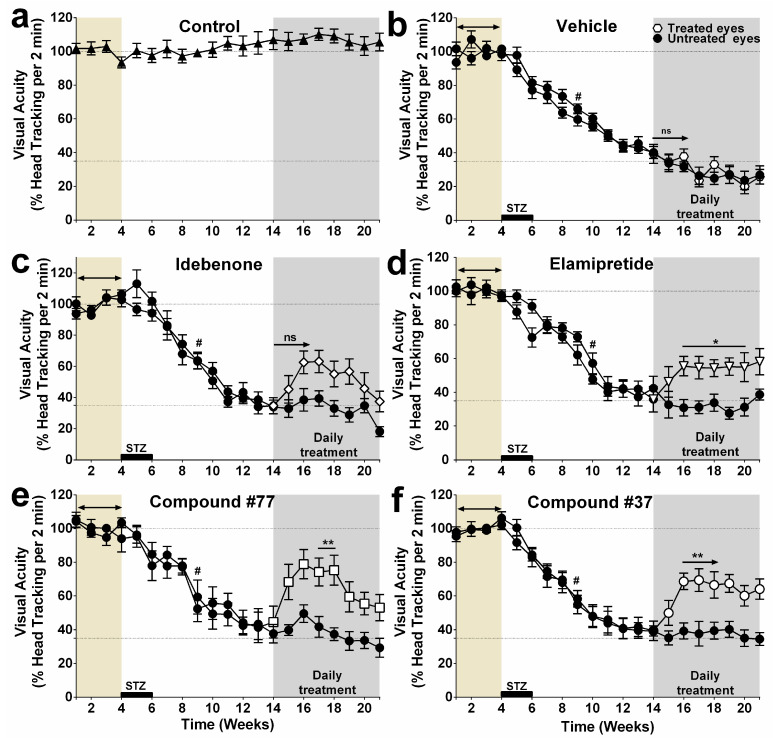
Effect of pharmacological intervention on visual function. Daily eyedrop administration was performed, from Week 14, with test compounds and short-chain quinones (SCQs), idebenone (10 mg/mL) (**c**), compound #77 (7.36 mg/mL) (**e**), compound #37 (4.6 mg/mL) (**f**), and elamipretide (26 mg/mL) (**d**), to the right eye of diabetic rats. Effects on visual acuity were quantified as percentage of head-tracking movements (optokinetic response (OKR)) and compared to untreated eyes (intra-animal control). The data represent mean (*n* = 8–12 eyes/group, *n* = 11 eyes/group for idebenone, *n* = 10 eyes/group and 9 eyes/group for #37- and #77-treated eyes, respectively, while *n* = 8 eyes/group for elamipretide-treated eyes, *n* = 11 eyes/group for Veh-treated group, and *n* = 12 eyes/group for healthy nondiabetic control (Cont) (see Supplementary Materials, Table S3 for details). Error bars = SEM, *p*# or *< 0.05, and ** *p* < 0.01 using two-way repeated measure ANOVA). Significant vision loss (#) as compared with baseline (average OKR for Weeks 1–4) were calculated for all weeks until Week 14. Significant therapeutic effects were calculated from Week 14 by comparing the OKR of treated against untreated eyes. ((**a**) Control, healthy nondiabetic control; (**b**) Vehicle, vehicle treated group; (**c**) Idebenone, idebenone treated group; (**d**) Elamipretide, elamipretide treated group; (**e**) Compound #77, #77-treated group; (**f**) Compound #37, #37-treated group, brown area, baseline observation period (Weeks 1–4); grey area, treatment period, ns – not significant).

Consistently, a significant decrease in visual acuity was detected by Week 9 (=5 weeks post-diabetes induction) (Figure 1b–f). Visual acuity continued to decline further until Week 14, when about 65% of vision loss was evident in both eyes of all diabetic animals as compared with their baseline value (*p* < 0.0001, Figure 1b–f). When visual function of the treatment groups (Figure 1b–f) was compared against nondiabetic rats (Figure 1a), visual acuity declined for all treatment groups between Weeks 9 and 14 (Figure 1a–f, graphs separated for clarity).

From Week 14 onwards, daily eyedrop administration of test compounds to the right eyes of diabetic rats were performed for 7 weeks (grey area, Figure 1b–f). Within 2–3 weeks, a significant improvement in head-tracking movements was observed for all test compound-treated groups, except for the idebenone-treated group (Figure 1b–f). Residual visual acuity increased between 23 and 45% in all test compound-treated groups. For #37- and elamipretide-treated animals, visual acuity in the right eyes was significantly restored after only 2 weeks of treatment, while the left untreated eyes continued to deteriorate. This effect was well preserved for 5 weeks and 4 weeks, respectively. In the #77-treated group, visual acuity of the treated eyes was not significantly different to the untreated eyes until 3 weeks of treatment (Figure 1e). Improved vision was sustained for only one week and, for the last 3 weeks of the study, no significant differences in visual acuity between treated and untreated eyes were detected for the #77-treated group. Despite a 30% increase in visual acuity in the idebenone-treated group, it did not reach significance as compared with the untreated eyes throughout the 7 weeks of treatment (Figure 1c).

**Figure 2:**
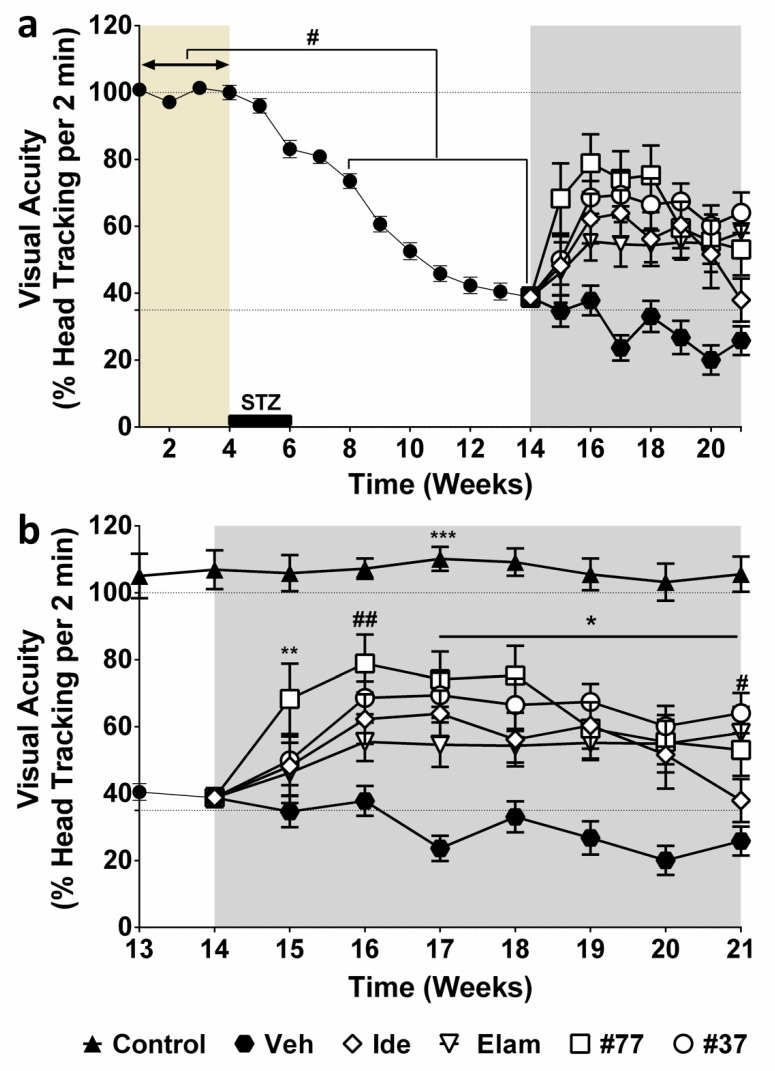
Statistical analysis of therapeutic effects of test compounds. (**a**) Combined OKR data of Figure 1. Data of all diabetic animals was pooled between Weeks 1 and 14 to provide a reliable course of vision loss. From Week 15, results for individual treatments are shown. (**a**) Significant vision loss (#) was calculated by comparing baseline values (average OKR for Week 1–4) against all weeks until Week 14. (**b**) Comparison of visual acuity of nondiabetic versus diabetic as well as treatment groups. Treatment with test compounds idebenone (Ide) (10 mg/mL), #77 (7.36 mg/mL), #37 (4.6 mg/mL), and elamipretide (Elam) (26 mg/mL) from Weeks 14 to 21 (grey area). Treatment effects were compared to vehicle-treated eyes (Veh), as well as against nondiabetic control. The data are expressed as mean (*n* = 49 eyes/group for untreated eyes (Weeks 1–14), *n* = 11 eyes/group for idebenone (Weeks 14–21), *n* = 10 eyes/group and 9 eyes/group for #37- and #77-treated eyes, respectively, (Weeks 14–21), while *n* = 8 eyes/group for Elam-treated eyes, *n* = 11 eyes/group for Veh-treated group, and *n* = 12 eyes/group for healthy nondiabetic control (Cont) (see Supplementary Materials, Table S3 for details). Error bars = SEM, (**a**) # *p* < 0.0001, (**b**) # or * *p* < 0.05, ** *p* or ## *p* < 0.01 and *** *p* < 0.0001 using two-way repeated measure ANOVA. From Week 14, test compound-treated eyes were compared against vehicle-treated eyes (*, #, **, ##); and test compound-treated eyes against healthy nondiabetic eyes (***). Baseline period (Weeks 1–4), brown area; double sided arrow represents average baseline OKR; treatment period (Weeks 14–21), grey area.

Subsequently, the data for the individual treatment groups were pooled and compared against the vehicle-treated group (Figure 2). In this analysis, all test compounds significantly improved vision, while the visual acuity of the vehicle-treated group (closed hexagon) did not improve (Figure 2b). This improvement was preserved for all test compounds over the observation period, except for idebenone (open diamonds) as compared with #37-treated animals (open circle), in which visual acuity significant declined in Week 21 (# *p* < 0.05) (Figure 2b). Compounds #37 and #77 dramatically improved vision within 2 weeks. Compound #77 (open squares) induced the fastest and highest recovery of head-tracking movements (** *p* < 0.01), restoring visual acuity from a residual ~35% in Week 14 to ~80% in Week 16. Although the #37-treated group appeared to show less vision recovery at Week 16 (up to ~65% of normal), there was no statistical difference between the #37- and #77-treated groups over the observation period. Despite being the least hyperglycemic group (Supplementary Materials, Figure S1), elamipretide-treated animals (open triangles) experienced the lowest recovery of visual acuity (both in extend and speed), where visual acuity was only restored to ~58% within 3 weeks of treatment. Although topical application of all test compounds restored visual function, it did not affect other parameters such as blood glucose levels, water intake, or body weight (other surrogate markers of diabetes) (grey areas in Supplementary Materials, Figure S1).

### 2.2. Short-Chain Quinones (SCQs) and Elamipretide Inhibit Diabetes-Induced Retinal Ganglion Cell Loss

**Figure 3:**
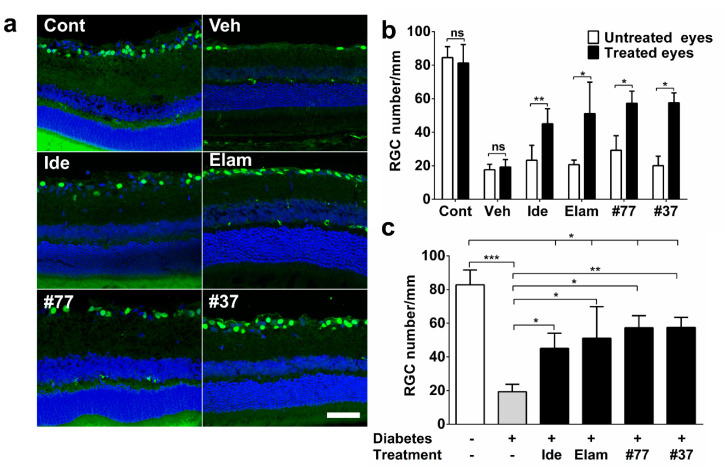
Effect of test compounds on retinal ganglion cell (RGC) numbers. (**a**) Representative images of Brn3a-stained retinal cryosections from nondiabetic control animals (Cont), as well as 5 treatment groups. Vehicle (Veh), idebenone (Ide), elamipretide (Elam), #77, #37 are shown. Scale bar = 100 µm. Images acquired at 20× magnification. Brn3a (green) and DAPI (blue); (**b**) Analysis of average numbers of RGC/mm of treated eye against untreated eye (intra-animal control) using ImageJ software. the Mann–Whitney test was used to compare treated eyes against untreated eyes for all treated groups; (**c**) Comparison of treatment groups against vehicle-treated (Veh) and nondiabetic group. The data represent mean for each treatment group (*n* = 6–12 eyes/group (3 independent samples), see Supplementary Materials, Table S2 for detail). Cont, nondiabetic control; Veh, vehicle; Ide, idebenone; Elam, elamipretide; ns, not significant. Significance was determined using the Kruskal–Wallis test. Error bar = SD. * *p* < 0.05, ** *p* < 0.01, *** *p* < 0.001.

All test compounds significantly protected against hyperglycemia-induced RGC loss as compared with vehicle-treated diabetic rats (*p* < 0.05) (Figure 3a–c). Treatment with #37 and #77 showed the best protection against RGC loss with only a 25% reduction in RGC /mm as compared with the untreated diabetic animals, (*p* < 0.05) (Figure 3c). This was followed by the elamipretide-treated eyes with a 40% loss in RGC numbers (*p* < 0.05) (Figure 3c). The idebenone-treated group showed a 45% reduction in RGC numbers, which was still significantly higher as compared with untreated diabetic rats (*p* < 0.05) (Figure 3c).

When RGC numbers from the treated eyes were compared with the untreated eyes (intra-animal control), no significant differences were observed in the vehicle-treated diabetic group (*p* > 0.05) (Figure 3b). In contrast, the treated eyes of all the test compound groups were significantly protected against RGC loss as compared with their respective untreated eyes (*p* < 0.05) (Figure 3b). When all the untreated eyes of the different groups were compared against each other, RGC numbers in the untreated eyes of the #77-treated group were unexpectedly higher as compared with the untreated eyes of the vehicle group (*p* < 0.05) (Figure 3b, significance icon not shown due to clarity).

### 2.3. SCQs and Elamipretide Protect Against Diabetes-Induced Retinal Thinning

**Figure 4:**
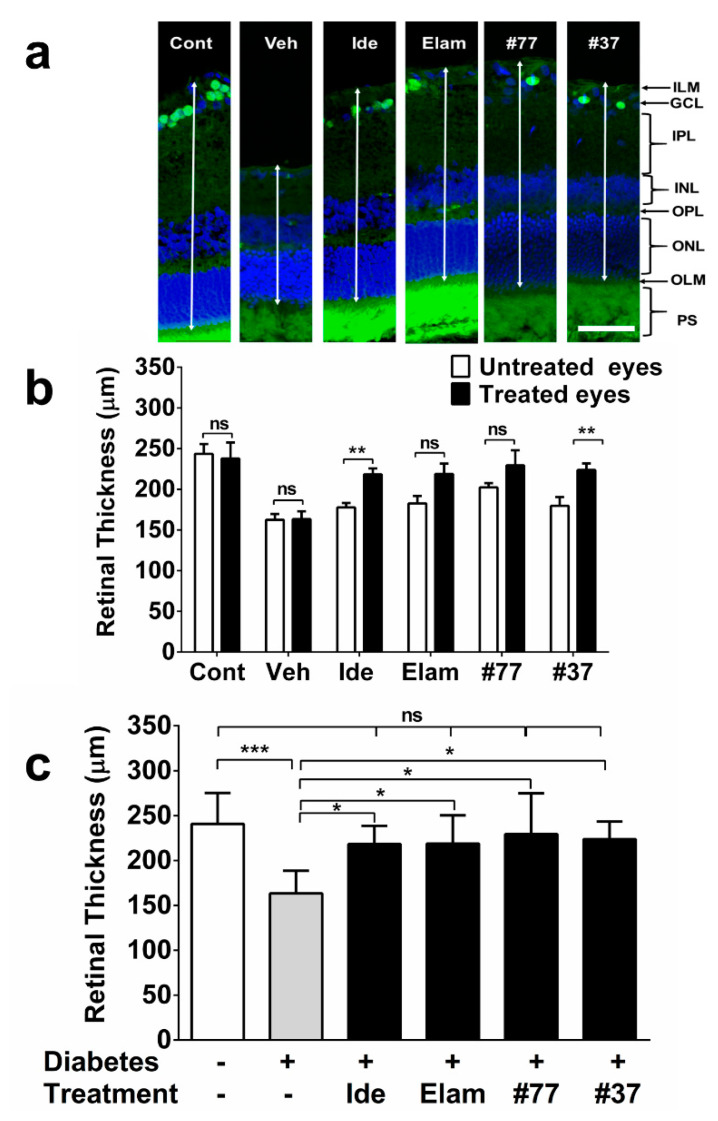
Effect of test compounds on retinal thickness. (**a**) Representative images of retinal cryosections from nondiabetic control animals, as well as 5 treatment groups. Scale bar = 100 µm. Images were acquired at 20× magnification. Brn3a (green) and DAPI (blue). Arrows exemplify measured retinal thickness between inner limiting membrane (ILM) to outer limiting membrane (OLM); (**b**) Quantification of retinal thickness by intra-animal comparison between treated and untreated eyes using ImageJ software. The data represent mean for each treatment group (n = 6–12 eyes/group (3 independent samples), see Supplementary Materials, Table S2 for detail). Significance was calculated using the Mann–Whitney test to compare treated (right) against untreated eyes (left); (**c**) Comparison of retinal thickness for all treatment groups against vehicle treated, as well as nondiabetic control. The data represent mean for each treatment group (*n* = 6–12 eyes/group, see Supplementary Materials, Table S2 for detail). Cont, nondiabetic control; Veh, vehicle; Ide, idebenone; Elam, elamipretide; ns, not significant. Significance was calculated using the Kruskal–Wallis test. Error bars = SD. * *p* < 0.05 ** *p* < 0.01, *** *p* < 0.001.

All test compounds significantly protected against hyperglycemia-induced retinal thinning as compared with the vehicle treated diabetic rats (*p* < 0.05) (Figure 4a–c), which was also illustrated by a lack of significant difference among the average retinal thickness in all test compound-treated eyes as compared with the healthy nondiabetic eyes (Figure 4c).

When treated eyes were compared against the untreated eyes within the groups (intra-animal control), no significant differences in retinal thickness were observed in the nondiabetic and vehicle-treated groups (*p* > 0.05) (Figure 4b). In contrast, the average retinal thickness in the treated eyes of two of the test compounds groups (idebenone and #37) was significantly increased (*p* < 0.01) as compared with the untreated eyes (Figure 4b). The difference in retinal thickness of the treated as compared with the untreated eyes for the elamipretide and #77-treated group did not reach statistical significance (*p* > 0.05) (Figure 4b). Similar to the RGC results, when the untreated eyes from all groups were compared against each other, a significant difference between the #77-treated group and the vehicle-treated diabetic group was observed (*p* < 0.05) (Figure 4b, significant icon not shown in graph for clarity). 

### 2.4. Novel SCQ Protects Against Diabetes-Induced Gliosis

**Figure 5:**
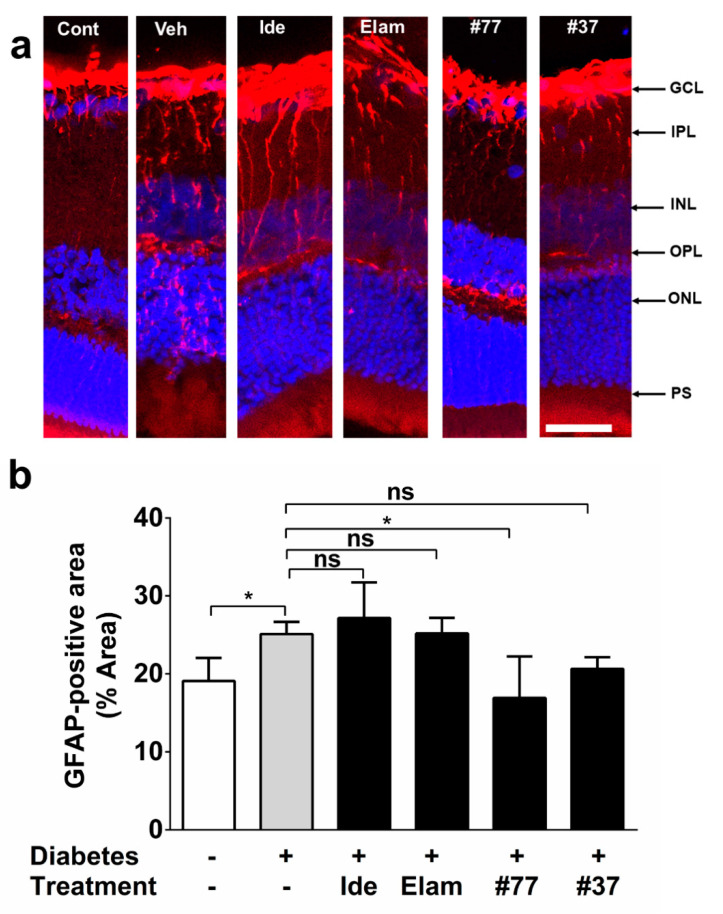
Effect of test compounds on reactive gliosis. (**a**) Representative images of retinal cryosections from nondiabetic (control) animals, as well as 5 treatment groups. Nondiabetic control (Cont), vehicle (Veh), Idebenone (Ide), elamipretide (Elam) #77 and #37. Images were acquired at 20×. Scale bar = 100 µm. GFAP (red) and DAPI (blue); (**b**) Quantification of test compound effects on reactive gliosis. GFAP-positive areas in the retina (% of total area) were quantified using ImageJ software and represent average GFAP-positive areas for all treatment groups against the vehicle-treated, as well as the nondiabetic (control) group. The data represent mean with *n* = 6 eyes/group (3 independent samples). Error bar = SD. * *p* < 0.05, ns – not significant (using the Kruskal–Wallis test). Significance labels of test compound groups against the nondiabetic control group are omitted for clarity.

In healthy, nondiabetic control retinas, glial fibrillary acidic protein (GFAP) reactivity was restricted to astrocyte and Muller cells in the retinal ganglion cell (RGC) layer (Figure 5a). In contrast, in diabetic rats, activated Muller cell projections extended from the inner plexiform layer (IPL) to the inner nuclear layer (INL) (Figure 5a). A significant increase in GFAP expression was detected in diabetic as compared with nondiabetic control animals (Figure 5b) (*p* < 0.05). After 7 weeks of treatment, only #77 had significantly reduced GFAP immunoreactivity (*p* = 0.0169), with no significant difference between the nondiabetic control and the #77-treated group (Figure 5b, significance is not shown in graph for clarity).

### 2.5. Novel SCQ Protects Against Diabetes-Induced Vascular Leakage

**Figure 6:**
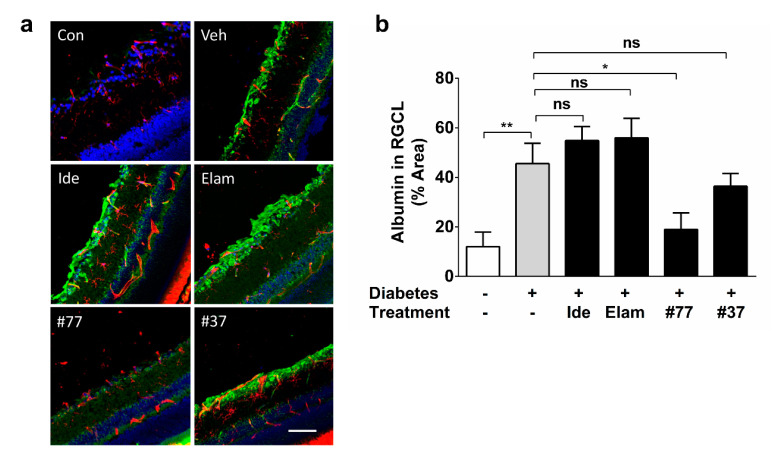
Effect of test compounds on retinal vascular leakage. (**a**) Representative images of retinal cryosections from nondiabetic control animals, as well as 5 treatment groups. Scale bar = 100 µm. Images were acquired at 20× magnification. Albumin (green), tomato lectin (red), and DAPI (blue); (**b**) Quantification of test compound effects on vascular leakage. The data represent average albumin-positive areas of all treatment groups against the vehicle-treated as well as the nondiabetic (control) group. The data represent mean with *n* = 5 eyes/group (3 independent samples). Error bars = SD. * *p* < 0.05, ** *p* < 0.01 (significance calculated using the Kruskal–Wallis test). Cont, nondiabetic control; Veh, vehicle; Ide, idebenone; Elam, elamipretide, ns, not significant. Significance labels of test compound groups against the nondiabetic control group are omitted for clarity.

Albumin immunoreactivity in the RGC layer was significantly increased in vehicle-treated diabetic as compared with healthy nondiabetic rats (*p* < 0.01) (Figure 6a,b). Albumin immunoreactivity was predominantly observed in the RGC layer; a weak signal was observed in the inner plexiform layer (IPL) and inner nuclear layer (INL), and, to a lesser degree, in the outer plexiform layer (OPL) and outer nuclear layer (ONL) (Figure 6a). Treatment with idebenone, elamipretide, and #37 did not show any significant effects as compared with the untreated diabetic group (*p* > 0.05). In contrast, #77 treatment significantly reduced albumin reactivity as compared with the untreated diabetic group (*p* < 0.05) (Figure 6b), while no significant difference was observed between the healthy control and the #77-treated groups.

### 2.6. Novel SCQs and Elamipretide Inhibit Diabetes-Induced Oxidative Stress

**Figure 7:**
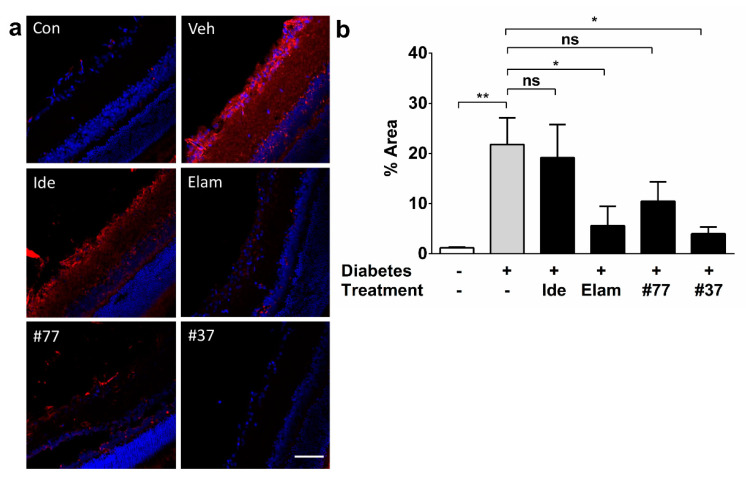
Effect of test compounds against oxidative protein damage in the retina. (**a**) Representative images retinal cryosections from nondiabetic control animals, as well as 5 treatment groups. Scale bar = 100 µm. Images acquired at 20× magnification. Nitro-tyrosine (red) and DAPI (blue); (**b**) Quantification of test compound effects on oxidative damage. The data represent average nitro-tyrosine-positive areas of all treatment groups against the vehicle-treated as well as the nondiabetic (control) group. The data represent mean with *n* = 5 eyes/group (3 independent samples). Error bars = SD. * *p* < 0.05, ** *p* < 0.01 (significance was calculated using the Kruskal–Wallis test). Cont, nondiabetic control; Veh, vehicle; Ide, idebenone; Elam, elamipretide, ns, not significant. Significance labels of test compound groups against the nondiabetic control group are omitted for clarity.

In order to correlate protection of visual acuity by the test compounds with additional markers of retinal histopathology, oxidative tissue damage was assessed by detecting 3-nitrotyrosine (Figure 7a,b). Immunoreactivity against 3-nitrotyrosine was predominantly observed in the RGC layer and to a lesser degree in the inner plexiform layer (IPL), inner nuclear layer (INL), outer plexiform layer (OPL), and outer nuclear layer (ONL) (Figure 7). Although immunoreactivity was detected in all retinal layers, a strong signal was mainly observed in the RGC layer of diabetic as compared with nondiabetic animals (*p* < 0.01) (Figure 7a). 

Both #37 and elamipretide significantly protected against oxidative damage as compared with vehicle-treated diabetic rats (*p* < 0.05) (Figure 7b) with no significant difference to the nondiabetic control animals (*p* < 0.05) (Figure 7b, significance icons for comparison of test compounds groups versus control group were omitted for clarity). Treatment with idebenone or #77 did not show any statistically significant reduction in oxidative damage as compared with the vehicle-treated diabetic group (*p* > 0.05).

## 3. Discussion

DR is typically diagnosed by continued ophthalmological monitoring as soon as diabetes is established and is based on the characteristic vascular pathology that current treatments focus on. Anti-VEGF antibodies have revolutionized the treatment of DR, especially in the proliferative stages as well as for the treatment of diabetic macular edema (DME) but require strict glycemic control [7]. However, no approved treatments address the early and mild nonproliferative stages of DR, while the landmark phase III RISE/RIDE trials reported increased visual acuity in about 45% of patients with DR/DME in response to anti-VEGF treatment [7,20], which highlights the need for additional treatment approaches. This study investigated if mitoprotective drugs could restore vision in a rodent model of DR. Side-by-side, the novel mitoprotective SCQs [18] restored vision to a greater extent than the reference compounds idebenone and elamipretide, while all compounds also protected against RGCs loss, retinal thinning, gliosis, vascular leakage, and oxidative stress, albeit with different efficacies. Overall, the novel SCQs consistently provided comparable or better protection of visual acuity and normalization of histological markers.

Increased levels of oxidative stress and apoptosis in DR have been linked to mitochondrial dysfunction [7,8,11,13]. This pathology affects both retinal neurons and vascular cells [8,13] by a vicious cycle where mitochondrial dysfunction activates inflammatory mediators and ROS production, which damages mtDNA, increases mitochondrial pathology, and eventually leads to apoptotic cell death [12]. Once activated, this cycle perpetuates the DR pathology and might explain why patients, despite well controlled blood glucose levels, are still at risk of DR. Consequently, therapies to improve this mitochondria-driven disease progression could be effective for counteracting the initiation and progression DR, which is supported by the current study. 

All test compounds rapidly improved visual function within 23 weeks, while a previous report showed improvement of vision using elamipretide in a mouse model of DR after 1 week, with full recovery after 12 weeks [8]. However, the diabetic animals, in the present study, displayed significantly worse visual function before treatment was initiated as compared with the previous study [8], likely as a consequence of significantly milder levels of hyperglycemia (~14 mmol/L) as compared with the present study (>25 mmol/L) [8]. Elamipretide, in the presence of a significantly more pronounced decline in visual acuity, also failed to achieve a full recovery of vision [8]. 

The observed hyperglycemia-induced RGC loss and reduced retinal thickness replicated previous preclinical and clinical studies where RGC loss was one of the earliest morphological signs of DR associated with reduced expression of brain-derived neurotrophic factor (BDNF) [21,22,23]. However, other studies found no evidence of RGC loss, even under conditions of chronic hyperglycemia [24]. This discrepancy may be due to different RGC detection methods, their quantification, animal strains used, and the degree of hyperglycemia achieved in the different studies [25,26]. For example, Brn-3a has been reported to only detect RGC loss after a relatively longer duration of hyperglycemia as compared with the use of the neuronal biomarker NeuN [27]. 

The present study challenges the dogma that vision loss is caused by RGC loss, and therefore should be irreversible. One plausible explanation can be derived from LHON and glaucoma as model diseases characterized by RGC loss, where the following three distinct stages were proposed: a pre-symptomatic, acute, and atrophic phase [27]. During the acute phase, vision loss occurs due to the interplay of mitochondrial dysfunction, decreased ATP production, and increased oxidative stress that results in dysfunctional but viable RGCs [28,29]. This phase has been proposed to be transient but, if not resolved, would subsequently develop into the atrophic phase, characterized by irreversible RGC death and optic nerve degeneration, when recovery of vision is no longer possible [28,30]. This model is largely supported by the clinical experience of LHON and glaucoma patients, and suggests a window of opportunity, possibly up to several years [30,31], where recovery of vision is still possible. Therefore, based on the protection of RGC numbers by the test compounds, in the present study, we likely initiated treatment in the acute phase of the disease. 

Although all test compounds have been described as mitoprotective and redox active [8,18], several distinct histological differences were observed. None of the compounds, except for #77, significantly prevented reactive gliosis, in contrast to a prior report where systemic idebenone prevented reactive gliosis in a mouse model of LHON [32]. This difference could be attributed to the timeline of GFAP upregulation that commences as early as 3 weeks after hyperglycemia and increases over time [33]. In the LHON model, treatment commenced concurrent to disease induction, while in the current study, treatment started only at Week 14, which might be too late to prevent GFAP overexpression by retinal Muller cells or astrocytes [34,35]. Although increased GFAP expression is indicative of a proinflammatory phenotype, the expression of inflammatory cytokines (such as Il-1β, Il-6, and TNF-α) and growth factors such as VEGF-A was not performed. The connection between mitochondrial dysfunction and the initiation and progression of inflammation has previously been established in many different disease models [36,37], therefore, this study aimed at focusing on the hypothesis that mitochondrial dysfunction is a major driver of vision loss in DR.

While #77 fully protected against vascular leakage, comparable to the healthy nondiabetic control, idebenone, elamipretide, and #37 were ineffective, which might suggest that #77 might be acting via different mechanism(s) as compared with the other test compounds. Since GFAP reactivity is a direct consequence of vascular leakage into the retina [35,38], both endpoints support our results for #77. In contrast to elamipretide and #37, #77 did not show a significant antioxidant effect, which suggests that the protection by #77 appears to involve mechanisms(s) distinct to the other compounds. Idebenone is widely portrayed as being a potent antioxidant [13] but, in the present study, there is no statistically significant antioxidant effect, which is in stark contrast to a previous report, where idebenone significantly reduced markers of oxidative damage in a rat model of cataract formation [39]. This effect was dependent on the route of administration and dose and, while the cataract study used oral idebenone (100 mg/kg), the present study used 10 mg/ml in eyedrops. The current study was not designed to assess compound concentrations in the vitreous, which is a limitation and future studies are planned to generate detailed pharmacokinetics (PK) profiles for the new SCQs. It is also important to highlight that the present study did not use the same concentrations of test compounds. Test compound concentrations based on weight would have falsified the interpretation of results, due to their significantly different molecular weights. Since the eyedrop vehicle did not allow for equimolar concentrations due to different solubilities, a possible solution could have utilized different vehicles for each test compound. Clearly, this approach would have made a side-by-side comparison questionable, which is why the test compounds were used at about, but not exactly, the equimolar dose of elamipretide. This is a clear limitation of the study, shared by most studies that attempt to do side-by-side comparisons, which in this case highlighted that the novel SCQs, although used at a lower concentration than elamipretide or idebenone, appeared to be significantly more protective.

In this study, both SCQs, idebenone and elamipretide, demonstrated several effects including antioxidant and retinoprotective effects associated with improved visual function. According to these results, three tests compounds (#37, idebenone, and elamipretide) appear to restore visual function primarily via neuronal pathways instead of the much-favored microvascular pathway, while #77 seems to normalize vascular integrity. Although, mitochondrial function was not directly assessed in this study, measurements of oxidative damage, which precedes and accompanies mitochondrial dysfunction and RGC apoptosis, were conducted [13,40]. While all test compounds protected retinal function, only one of them normalized retinal vascular integrity. This could either suggest that therapeutic strategies for certain stages of DR do not necessarily have to include normalization of vasculature or that mitoprotective agents could be used in conjunction with vascular therapies. Certainly, mitoprotective agents that also protect retinal vascular integrity could be promising development candidates. In contrast, protection against oxidative retinal damage, presumably by normalizing mitochondrial function could represent a promising mechanism by which at least #37 and elamipretide restore vision. According to the low expected concentrations of these compounds in the retina, a direct antioxidant function appears to be unlikely. Therefore, the actual molecular target of these compounds for exerting their antioxidant and retinoprotective effects still remains unclear. For idebenone, in addition to is presumed antioxidant function, direct interactions with the signaling molecules p52Shc and PPARα/γ have been reported to be relevant for its protective activity under diabetic conditions [41,42,43,44]. Both of these targets also involve the downstream activation of Akt that is known to inhibit mTOR signaling, which is a potential target of DR therapy [45]. Recently, another retinal mode of action has been proposed for idebenone that centers around the increased expression of Lin28a under hypoxic conditions in the retina [46,47]. In a rat model of retinal reperfusion injury, idebenone-induced overexpression of Lin28a restored visual acuity, and protected against retinal injury. Overexpression of Lin28a in a separate study induced a robust and sustained regeneration of the optic nerve in mice, which was also mediated by activation of Akt kinase [46]. It is intriguing to speculate that in DR, the presence of vascular abnormalities could lead to a hypoxic state [1], that could enable this idebenone-dependent pathway. However, we did not find any histological evidence for Lin28a upregulation in our test animals (data not shown) and can only speculate that due to the length of our treatment protocol this effect might no longer have been visible.

Overall, the present study demonstrates that the potential of mitoprotective SCQs should be explored further as novel therapeutic options against DR and other related ophthalmological indications. However, the detailed mode of action for the new SCQs remains unclear and will be the topic of future investigations to assess the therapeutic potential and risks of this class of compounds.

## 4. Materials and Methods 

### 4.1. Test Compounds and Formulation

Idebenone was provided by Santhera Pharmaceuticals (Pratteln, Switzerland). The SCQs #37 and #77 as well as elamipretide were synthesized and confirmed for purity of >95% using nuclear magnetic resonance (NMR) spectroscopy and mass spectrometry (MS), as described previously [18]. All compounds were stored in powder form, at 4 °C. Test compounds were dissolved in eyedrop solution (5% tyloxapol, 5% mineral oil in 66 mM citrate buffer, pH 7.4). Eyedrop solution without test compounds served as vehicle. All other chemicals were obtained from Sigma-Aldrich (Castle Hill, NSW, Australia), if not stated otherwise.

### 4.2. Animals

Male Long–Evans rats (aged 23–24 weeks, average body weight of 392.11 ± 23.12 g) were used in this study, in accordance with the Australian Code for the Care and Use of Animals for Scientific Purposes (8th edition, 2013) [48] with approval from the University of Tasmania Animal Ethics Committee (A0016524, 23 June 2015). All rats used for this research were obtained from the University of Tasmania (Hobart, Australia) animal facility, the Monash Animal Research Platform (MARP, Melbourne, Australia), or the Animal Resources Centre (ARC, Perth, Australia). Animals were acclimatized for 7 days before the study commenced. Animals were randomized and housed in groups of three at 21 ± 2 °C, with a 12 h/12 h light-dark cycle using a 2000 cm^2^/cage rodent housing system (Allentown Inc., Allentown, NJ, USA). All cages were enriched with bedding materials, dark nest boxes, small wooden sticks for gnawing, autoclaved tissues, and chewable small toys. Initially, cage bedding and bedding materials were frequently changed once or twice a week, which was increased to every second day when diabetic rats experienced polyuria. The health of rats was monitored daily across the experimental protocol.

### 4.3. Measurement of Food, Water-Intake, and Body Weight

Food and water were provided ad libitum throughout the study for all animals, as previously reported [17]. All rats were provided with both chow and high fat diet (HFD, 40% energy from fat, simple carbohydrate replacement, Specialty Feeds, WA, Australia). Body weights of test animals were measured using a digital balance (Albi Import, VIC, Australia). Water intake was monitored throughout the study as well.

### 4.4. Blood Glucose Measurement

Non-fasting blood glucose determination (BGL) was performed, as described previously [17]. BGL was measured weekly, using commercially available handheld glucometer (Accu-Chek^®^ Performa, NSW, Australia).

### 4.5. Induction of Diabetes Using STZ via Osmotic Minipump

**Figure 8:**
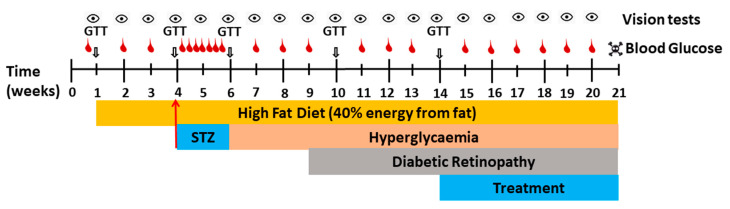
Schematic representation of study protocol. Rats received high fat diet (HFD) at the start of the observation period. Baseline parameters (bodyweight, blood glucose levels (red icons), water intake, and visual acuity (eye icon)) were monitored weekly over 3 weeks. At Week 4, animals received streptozotocin (STZ) via a minipump and daily blood glucose levels were monitored until pumps were removed at Week 6. Weekly parameters were continuously monitored to establish hyperglycemia and vision loss (diabetic retinopathy) throughout the study period. Daily treatment with test compounds started from Week 14. Intermittent glucose tolerance tests (GTT) were carried out to monitor insulin resistance. At the end of the observation period (Week 21) animal were culled.

Type 2-like diabetes was induced in all test animals (except the healthy control animals) by a combination of HFD and osmotic mini-pump (Alzet Model 2ML2, Durect Corporation, Cupertino, CA, USA) infused streptozotocin (STZ), as previously described [17]. Briefly, HFD was utilized to induce insulin resistance [49] and STZ dose-dependently depleted pancreatic beta cells [17]. Using a surgical scalpel (Swann Morton, Sheffield, England), an approximately 1.5 cm incision in length was made on the dorsal part of the rat around the lumbar region of the spinal cord, and the minipumps inserted. The minipumps delivered STZ (125 mg/kg in 0.1 M citrate buffer, pH 4.4) subcutaneously at a constant rate of 5 µL/h over a 14-day period [17]. As soon as blood glucose levels reached consistent hyperglycemic levels or 14 days after implantation, the pumps were removed to cease the delivery of STZ (Figure 8).

### 4.6. Treatment with Test Compounds

Prior to commencing drug treatment (Week 14, Figure 8), a second randomization was performed to ensure that all groups contained animals representing similar ranges of bodyweight, age, blood glucose level, and displayed a similar degree of vision loss. From Week 14 onwards, the right eyes of the diabetic rats were treated once daily (except for the control group) with/without test-compound-containing eyedrop solution until Week 21 (Figure 8). The left (untreated) eye served as intra-animal control. Test compounds were used at their respective solubility limits in eyedrop solution (0.03 M idebenone, 0.01 M #37, 0.02 M #77, and 0.03 M elamipretide).

### 4.7. Assessment of Visual Acuity in Rats Using Optokinetic Response

Visual acuity was based on optomotor behavior and measured weekly, as described previously [50,51,52]. An optokinetic reflex system was used to detect abnormalities in visual acuity (a measure of percentage (%) head-tracking movement per unit time or optokinetic reflex (OKR) [32]. Visual acuity of both the left and right eyes of all animals was assessed once weekly throughout the study period of 21 weeks. Briefly, after 2–3 min of adaptation to the apparatus, visual acuity testing was performed by clockwise and counter-clockwise rotation of the drum at an angular speed of 2.61 rpm for 2 minutes in each direction, with an interval of 30 seconds between the two rotations. Animal behavior was recorded with a HD 1080p webcam (Logitech^®^ C920 Carl Zeiss Tessar Dell, NSW, Australia) and subsequently scored in an investigator-blinded manner. For total number of animals use in the behavioral study see Supplementary Materials, Table S3.

### 4.8. Immunohistochemistry

Retinas collected at the end of the observation period (Week 21) were used to generate cryosections for histological examination using standard methods, as described previously [32,53]. After perfusion fixation (4% paraformaldehyde (PFA) in 0.1 M phosphate-buffered saline (PBS)) eyes were immediately collected and post-fixed in 4% PFA in 0.1 M PBS for 24 h, at 4 °C. Subsequently, eyes were transferred to 30% sucrose in 0.1 M PBS at 4 °C, for 24 h. Eyes were immersed in OCT compound (Tissue-Tek^®^, Sakura Finetek, Tokyo, Japan) before transferred, oriented, and placed in fresh OCT in cryomolds (15 × 15 × 5 mm) (Tissue-Tek^®^, Sakura Finetek, Tokyo, Japan). Tissues were slowly frozen in liquid nitrogen and stored at −80 °C before sectioned with a cryotome (Leica CM1850 UV, Leica Microsystems, VIC, Australia) along the sagittal-horizontal axis into 20 µm sections. Sections were transferred to 24 × 50 mm IHC microscope glass slides (Dako, NSW, Australia), and dried at room temperature overnight, before stored at 4 °C.

For immunostaining, sections were blocked in 1% BSA, 22.52 mg/mL glycine, in 1x PBS + 0.1% Tween 20, for 1 h at room temperature. Tissue sections were incubated overnight at 4 °C with primary antibodies against Brn-3a, GFAP, nitro-tyrosine, albumin, while fluorescein-labelled tomato lectin was used to visualize blood vessels (Supplementary Materials, Table S1). Primary antibodies were detected with species-specific fluorophore-conjugated secondary antibodies (Supplementary Materials, Table S1), while DAPI (1:200,000, Invitrogen, VIC, Australia) was used to stain nuclei. Sections were mounted using fluorescence mounting media (Dako, NSW, Australia) and stored at −20 °C for imaging. All histological data were assessed from an average of 5–12 sections per treatment group (Supplementary Materials, Table S2). Sections with obvious retinal damage from procedural processes such as cutting artefacts, tissue tearing, and folding were excluded from the analysis.

### 4.9. Image Acquisition

Images were acquired at 20× magnification using a confocal microscope (NIS-Elements AR software version 5.02.00, Nikon Eclipse Ti, Nikon, SA, Australia) with a CMOS camera (Andor Zyla, Oxford Instrument, Abingdon, UK), as described [53]. For the quantification of retinal histological features, 2147 × 1208 pixel images were acquired from three different region of the retina, as described [53,54]. The number of RGCs (RGCs per mm) in the ganglion cell layer (GCL) were counted for the entire captured retinal image using imageJ software (version 1.8) (National Institutes of Health, Bethesda, MD, USA). Total retinal thickness (TRT) was quantified in each image at 3 points and averaged for each image before subsequently averaged across the images of each treatment group, as reported in [55]. Retinal thickness was measured from the inner limiting membrane (innermost border of the GCL) to the outermost border of the photoreceptor layer (PL). Reactive gliosis was quantified by scoring the extent of GFAP staining (threshold intensity) using imageJ software, as described [55], which measured the percentage of GFAP-positive area in the inner plexiform (IPL) and the inner nuclear layer (INL). GFAP expression in the ganglion cell layer (GCL), which is common in both diabetic and nondiabetic retinas, was excluded from the analysis. At least three separate retinal regions were analyzed for each eye. Identical thresholding parameters were applied to all images. Vascular leakage and oxidative damage were also measured using similar methods. For vascular leakage and oxidative damage, the GCL was included (see Supplementary Materials Table S2 for details).

### 4.10. Exclusion Criteria

During the study, some rats developed severe ketoacidosis during the experimental protocol and were culled prematurely due to ethical reasons. Animals culled before Week 18 were excluded from the behavioral study (see note under Supplementary Materials Table S3). Sections with obvious retinal damage from procedural processes such as cutting artefacts, tissue tearing, and folding were also excluded from the histological analysis.

### 4.11. Statistical Analysis

Data were expressed as mean ± standard error of mean (SEM) or ± standard deviation where applicable. Statistical and graphical presentations of data were performed using GraphPad Prism (Version 6, GraphPad Software Inc., San Diego, CA, USA). Statistical significance was calculated using the Kruskal–Wallis test for group differences at a single time point or two-way analysis of variance (ANOVA) for all time-course related comparisons, followed by multiple comparison tests to evaluate the differences between groups (that pass normality test). For Figure 3b and Figure 4b, the Mann–Whitney test was conducted. 

## Data Availability

The data presented in this study are available in the article or Supplementary Material.

## Supplementary Materials

Supplementary materials can be found at https://www.mdpi.com/1422-0067/22/3/1016/s1, Table S1: List of primary and secondary antibodies used for immunohistology, Table S2: Total eyes per treatment group used for immunohistochemistry, Table S3: Animal included in behavioral study over the 21 week period, Figure S1: Effect of STZ on blood glucose levels.
