# Supplementary material for: Novel Short-Chain Quinones to Treat Vision Loss in a Rat Model of Diabetic Retinopathy
Source: Int J Mol Sci. 2021 Jan 20;22(3):1016. doi: 10.3390/ijms22031016 (PMC7864174; doi:10.3390/ijms22031016)
Supplement: Supplementary file 1 [file ijms-22-01016-s001.pdf]

Supplementary Data

Table S1: List of primary and secondary antibodies used for immunohistology

**Product name/Catalogue no.**	**Company**	**Dilution**	**Reference no./Notes**
Mouse-anti-Brn-3a, (MAB 1585)	Merck Millipore (EMD Millipore), Bayswater, VIC, Australia	1:200	Lot: 2943286
Donkey-anti-mouse-Alexa Fluor 488, (ab150105)	Abcam, Melbourne, VIC, Australia	1:1000	Order no: 3288156
Rabbit-anti-Albumin, (GTX102419)	Sapphire Bioscience (GeneTex Inc), Redfern, NSW, Australia	1:500	Lot: 42312
Tomato Lectin, (DL-1177)	Abacus DX (Vector Laboratories), Meadowbrook, QLD, Australia	1:1000	
Goat-anti-GFAP, (ab53554)	Abcam, Melbourne, VIC, Australia	1:500	Lot: GR295831-2
Donkey-Anti-Goat-Alexa Fluor 568, (A11057)	ThermoFisher Scientific, Scoresby, VIC, Australia	1:1000	Lot: 1711491
Mouse-anti-3-nitro-tyrosine, (ab61392)	Abcam, Melbourne, VIC, Australia	1:1000	Clone no: 39B6

Table S2: Total eyes per treatment group used for immunohistochemistry

Test Compounds	RGC count (n)	Retinal thickness (n)	Reactive Gliosis (n)	Vascular leakage (n)	Oxidative damage (n)
Control	12	12	6	5	5
Vehicle	7	7	6	5	5
Idebenone	8	8	6	5	5
Elamipretide	6	6	6	5	5
#77	6	6	6	5	5
#37	6	6	6	5	5

Table S3: Animals included in behavioural study over a 21-week period*

Treatment	Up to Week 18	Up to Week 21	Total animal numbers
**Healthy control**	0	6	6
**Vehicle**	1	10	11
**Idebenone**	3	8	11
**Elamipretide**	0	8	8
**#77**	0	9	9
**#37**	0	10	10
**Total**	4	51	**55**

* Only the animals that were used for the analysis are listed in the table. 19 animals were culled prematurely (before the start of the treatment) due to ethical reasons, because of poor health. These animals are not listed in the table and were not used for the analysis. Those culled in week 18 were included in the analysis.

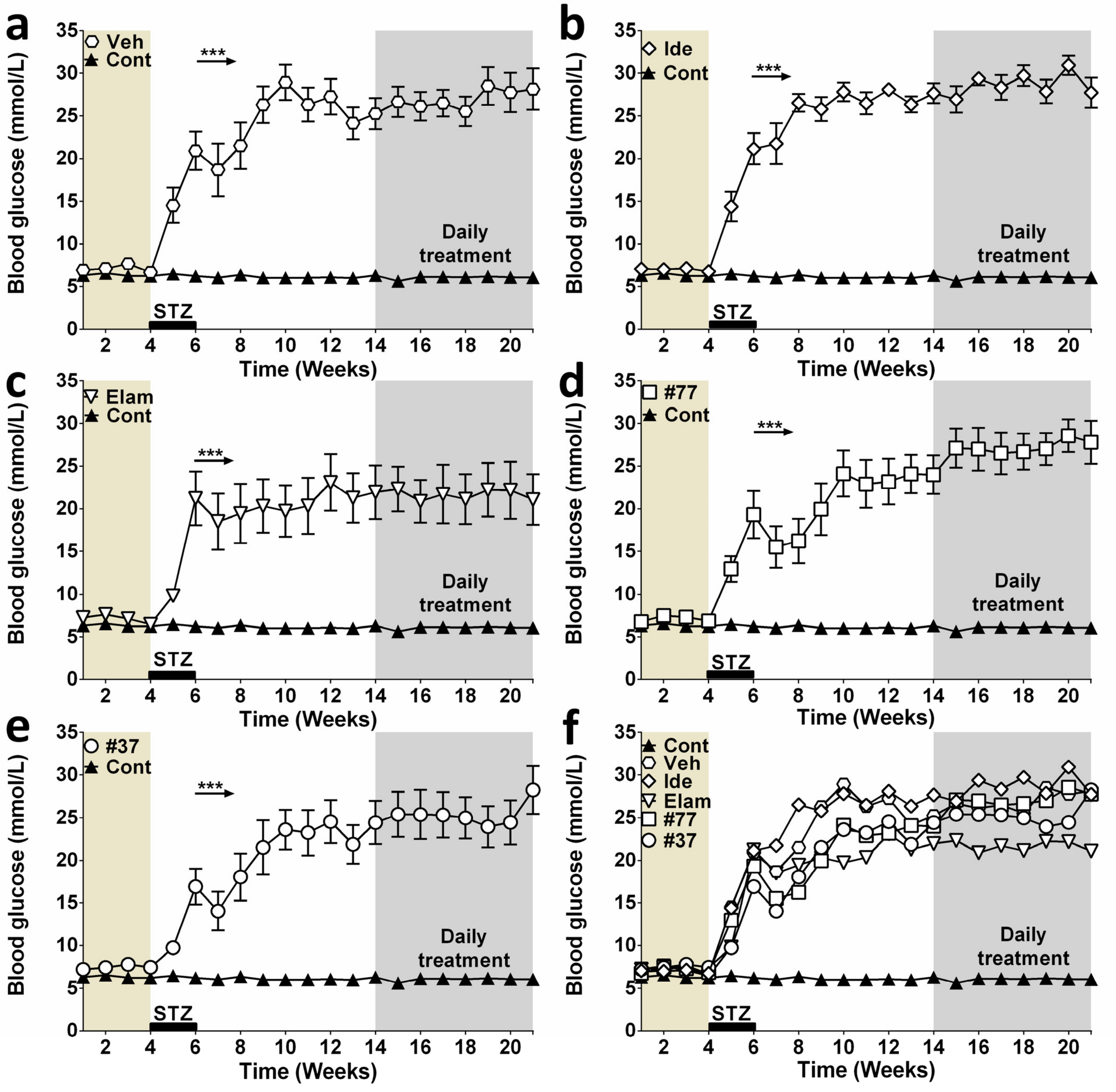

Figure S1: Effect of STZ on blood glucose levels

STZ (125mg/kg) was administered over a period of 2 weeks (starting from week 4) via osmotic pumps. (a-e) show the average weekly blood glucose level measurement for all five treatment groups of Long Evans rats when compared against the healthy non-diabetic group (control). (f) represent a pool of all data together. Data is expressed as mean (n = 8-11 rats/group, see Table S3 for detail) Error bar = SEM, p***< 0.001, using two-way ANOVA measures. Brown area: baseline observation period (weeks 1-4), grey area: treatment period. Error bars were omitted from (f) for clarity.
